# Iatrogenic Delayed Pneumothorax After Transbronchial Biopsy

**DOI:** 10.1177/2324709620947634

**Published:** 2020-08-05

**Authors:** Kartikeya Rajdev, Dustin N. Herring, Shubham Lahan, Kyle Wilson, Peter J. Murphy

**Affiliations:** 1 University of Nebraska Medical Center, Omaha, NE, USA; 2 University College of Medical Sciences, New Delhi, India

**Keywords:** pneumothorax, iatrogenic, delayed complication, transbronchial biopsy

## Abstract

Transbronchial biopsy (TBB) is one of the commonly performed procedures by pulmonologists in everyday practice. Although the procedure has a very low-risk profile, complications often develop in certain patients. Pneumothorax is one such complication pertaining to TBB. As only a small percent of procedures get complicated by pneumothorax, handful of cases have been reported with its delayed occurrence in the past 5 decades. The purpose of our report is to highlight another uncommon yet interesting case of delayed iatrogenic pneumothorax in an immunocompromised patient after TBB. Although the chain of events behind the pathophysiology of delayed pneumothorax largely remain a mystery, its development has been linked to altered immune mechanics as they are frequently recognized in immunocompromised patients.

## Introduction

Transbronchial biopsy (TBB) is a procedure routinely performed by pulmonologists for diagnosing conditions such as sarcoidosis, infections, and cancerous etiologies. Under rare circumstances, it can lead to pneumothorax as its potential complication. The incidence of pneumothorax following a TBB ranges from 1% to 5%.^1-4^ While pneumothoraces occurring shortly after a TBB are suspected and diagnosed readily, serious problems may arise when a pneumothorax presents as a delayed complication—defined as pneumothorax presenting 4 hours after a TBB. The reported incidence of these delayed pneumothoraces varies from 1% to 4%.^5-7^ In our case report, we acknowledge a case of delayed pneumothorax that came to recognition about 22 hours following TBB.

## Case Description

A 39-year-old man presented with worsening shortness of breath. The patient had a past medical history significant for follicular lymphoma (diagnosed 3 years ago) for which he had completed chemotherapy. He had a relapse of follicular lymphoma, which continued to progress despite chemotherapy with rituximab, cyclophosphamide, hydroxydaunorubicin, vincristine, and prednisone (R-CHOP); eventually received an allogenic stem-cell transplant 1 year ago. The posttransplant course was complicated by graft-versus-host disease of the gastrointestinal tract and skin, cytomegalovirus viremia, BK virus-associated hemorrhagic cystitis, varicella-zoster dermatitis, polymicrobial blood stream infections, and hypogammaglobinemia. He was being treated with oral budesonide, ruxolitinib, and tacrolimus for graft-versus-host disease. The patient was a former smoker with 5 pack-year smoking history with no other primary lung diseases such as chronic obstructive pulmonary disease, bronchiectasis, emphysema, or pulmonary fibrosis. His pulmonary function test showed a normal spirometry and lung volumes with mildly reduced DLCO (carbon monoxide diffusing capacity).

One month prior to this presentation, computed tomography (CT) scan of his chest showed multifocal pulmonary nodules suggesting invasive fungal infection. The patient was started on AmBisome and isuvaconazole and scheduled for bronchoscopy with TBB. Multiple TBB were performed in the lateral-basal segment of the right lower lobe of the lung. The procedure was performed without any complications, but no infectious etiology was identified, and the patient was discharged.

**Figure 1.**
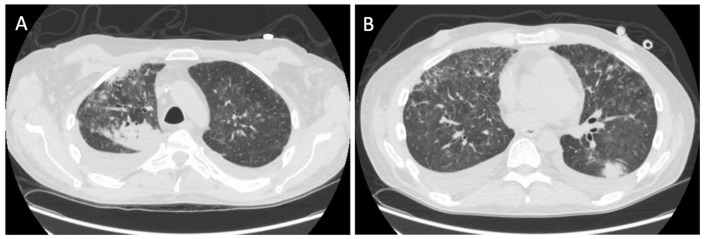
Computed tomography scan showing multifocal nodular opacities in the right upper lobe of lung (A) and the left lower lobe of lung (B).

**Figure 2.**
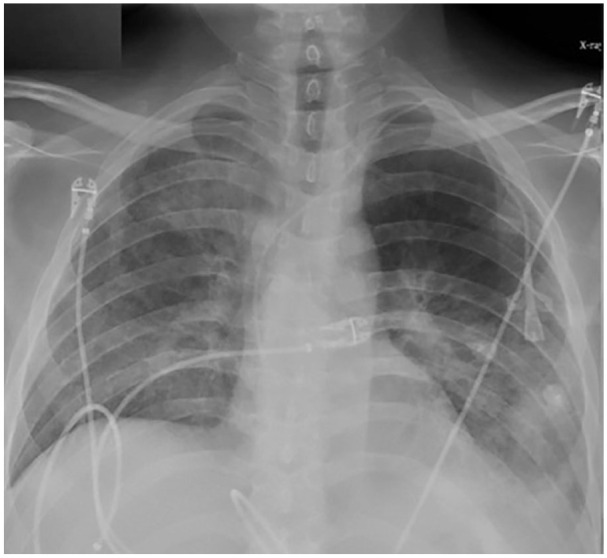
Initial chest X-ray obtained 35 minutes after bronchoscopy with transbronchial biopsy in the semi-upright position.

**Figure 3.**
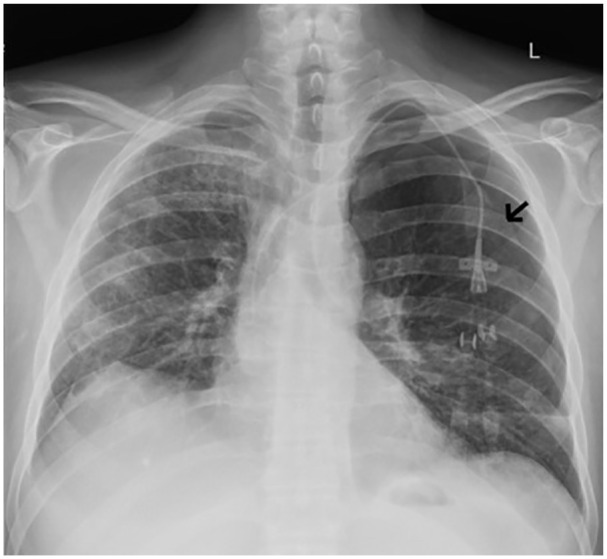
Follow-up chest X-ray performed when the patient was symptomatic (22 hours after transbronchial biopsy). Figure shows the presence of a left-sided apical pneumothorax (black arrow delineates the pleural line).

When he presented again to the hospital with worsening shortness of breath, a repeat chest CT was ordered, which in comparison with the CT scan 1 month ago, showed variably changed pulmonary opacities, with new/increased areas of more focal consolidation the right upper lobe (Figure 1A) and left lower lobe (Figure 1B), and with decreased nodular and ground glass opacities elsewhere in the lungs. We repeated the bronchoscopy with TBB—obtaining a total of 7 biopsies from the lateral-basal segment of the left lower lobe of the lung. Chest X-ray (CXR) obtained 35 minutes after the procedure was negative for pneumothorax (Figure 2). Twenty-two hours postprocedure, the patient complained of acute onset of left-sided pleuritic chest pain. CXR revealed a small apical pneumothorax that remained unchanged on serial CXR evaluations and the patient was discharged home. A follow-up CXR performed the following day on an outpatient basis revealed a worsening pneumothorax (Figure 3) and the patient was re-admitted for further management. A 12-French pigtail chest tube was placed under ultrasound guidance that led to the resolution of pneumothorax. The patient was discharged home after a hospital stay of 4 days.

## Discussion

Pneumothorax is an uncommon but known complication of TBB. Rarely, it can present as a delayed complication; however, time interval to its presentation often varies remarkably.^2,3^ Narula et al described a case where the patient was reported to present with respiratory distress and was diagnosed with iatrogenic pneumothorax secondary to TBB as late as 7 weeks after undergoing a TBB.^8^ Their patient had a past medical history of germ-cell tumor and persistent pulmonary infiltrates refractory to multiple courses of antibiotics. Kwan et al illustrated another similar case in which the patient (18 months post-lung transplant) developed delayed pneumothorax 5 months after TBB.^9^ Table 1 provides an overview of reported cases of delayed pneumothoraces following transbronchial biopsies.

**Table 1.** Overview of Reported Cases of Delayed Pneumothoraces After TBBs and Patient Characteristics.

Authors	Year	Time to presentation after TBB	Patient’s underlying conditions
Levy et al^3^	1986	• 36 hours • 5 days	• 15 years old; post renal transplant; PCP pneumonia. • 28 years old; 3-month history of weight loss; sputum negative, biopsy-positive *Mycobacterium tuberculosis*
Kwan et al^9^	2013	• 5 months	• 26 years old; 18 months post lung transplant due to cystic fibrosis; complicated by cryptogenic pneumonia and acute rejection; on 10 mg prednisone daily
Narula et al^8^	2018	• 7 weeks	• 62 years old; history of germ cell tumor and persistent LLL infiltrates; multiple courses of antibiotics without resolution; TBB showed lymphocytic predominance
Rajdev et al (current article)	2020	• 22 hours	• 39 years old; history of follicular lymphoma; post stem cell transplant; GVHD; CMV; VZV dermatitis; treated with budesonide, tacrolimus, ruxolitinib

Abbreviations: TBB, transbronchial biopsy; PCP, *Pneumocystis carinii* pneumonia; LLL, left lower lobe; GVHD, graft-versus-host disease; CMV, cytomegalovirus; VZV, varicella zoster virus.

The exact mechanism behind this delayed occurrence is yet to be determined. Levy et al hypothesized that TBB procedure results in the formation of a bronchopleural fistula that gets secured by a temporary fibrin clot formation. When this fibrin plug undergoes fibrinolysis gradually over days, it can cause a delayed pneumothorax as a result of air egressing through the defect.^3^ But no concrete evidence exists to support their hypothesis. Other proposed mechanisms and associated risk factors that are known to contribute to the development of delayed pneumothorax include absence of emphysematous changes in the lung parenchyma, persistence of a tissue flap after biopsy (obstructing the air flow), and microbial seeding through the puncture site.^1,9^

Interestingly, majority of these delayed presentations of pneumothoraces are attributed to altered immune mechanics (typically seen in immunocompromised patients) and poor wound healing—as repeatedly evidenced by their occurrences in organ transplant recipients and tuberculosis patients.^3,9,10^ The experience we gained from our patient scenario also suggests that immunologically weak patients are more prone to this serious complication of TBB.

The guidelines laid down by the British Thoracic Society recommend obtaining a post-biopsy CXR in symptomatic patients and that patients be advised of potential delayed complications of TBB.^11^ Ahmad et al recommend that 1-hour observation after TBB prior to obtaining a CXR may seem reasonable in outpatients, although complications rarely arise in this subset.^12^ Izbicki et al based on their level of evidence suggest that performing CXRs in asymptomatic patients after routine TBB adds minimal diagnostic value and avoiding them can be considered safe.^4,13^ However, there are no strict guidelines that mandate meticulous attention to duration (for which a patient should ideally be observed) or any “high-risk” patient characteristics so as to minimize the occurrence of delayed pneumothoraces.

We are of the opinion that physicians should remain cautious about patients undergoing transbronchial biopsies; moreover, patients should be educated about the possible risk of delayed pneumothoraces; and encouraged to seek immediate medical attention should they develop symptoms.
